# Music-performance regulates microRNAs in professional musicians

**DOI:** 10.7717/peerj.6660

**Published:** 2019-03-29

**Authors:** Preethy Sasidharan Nair, Tuire Kuusi, Minna Ahvenainen, Anju K. Philips, Irma Järvelä

**Affiliations:** 1 Department of Medical Genetics, University of Helsinki, Helsinki, Finland; 2 DocMus Doctoral School, Sibelius Academy, University of the Arts, Helsinki, Finland

**Keywords:** MicroRNA, Sequencing, Music-performance, Gene regulation, Peripheral blood, Cognition, Perception, Neuroplasticity

## Abstract

Musical training and performance require precise integration of multisensory and motor centres of the human brain and can be regarded as an epigenetic modifier of brain functions. Numerous studies have identified structural and functional differences between the brains of musicians and non-musicians and superior cognitive functions in musicians. Recently, music-listening and performance has also been shown to affect the regulation of several genes, many of which were identified in songbird singing. MicroRNAs affect gene regulation and studying their expression may give new insights into the epigenetic effect of music. Here, we studied the effect of 2 hours of classical music-performance on the peripheral blood microRNA expressions in professional musicians with respect to a control activity without music for the same duration. As detecting transcriptomic changes in the functional human brain remains a challenge for geneticists, we used peripheral blood to study music-performance induced microRNA changes and interpreted the results in terms of potential effects on brain function, based on the current knowledge about the microRNA function in blood and brain. We identified significant (FDR <0.05) up-regulation of five microRNAs; hsa-miR-3909, hsa-miR-30d-5p, hsa-miR-92a-3p, hsa-miR-222-3p and hsa-miR-30a-5p; and down-regulation of two microRNAs; hsa-miR-6803-3p and hsa-miR-1249-3p. hsa-miR-222-3p and hsa-miR-92a-3p putatively target FOXP2, which was found down-regulated by microRNA regulation in songbird singing. miR-30d and miR-222 corroborate microRNA response observed in zebra finch song-listening/learning. miR-222 is induced by ERK cascade, which is important for memory formation, motor neuron functions and neuronal plasticity. miR-222 is also activated by FOSL1, an immediate early gene from the FOS family of transcriptional regulators which are activated by auditory-motor stimuli. miR-222 and miR-92 promote neurite outgrowth by negatively regulating the neuronal growth inhibitor, PTEN, and by activating CREB expression and phosphorylation. The up-regulation of microRNAs previously found to be regulators of auditory and nervous system functions (miR-30d, miR-92a and miR-222) is indicative of the sensory perception processes associated with music-performance. Akt signalling pathway which has roles in cell survival, cell differentiation, activation of CREB signalling and dopamine transmission was one of the functions regulated by the up-regulated microRNAs; in accordance with functions identified from songbird learning. The up-regulated microRNAs were also found to be regulators of apoptosis, suggesting repression of apoptotic mechanisms in connection with music-performance. Furthermore, comparative analyses of the target genes of differentially expressed microRNAs with that of the song-responsive microRNAs in songbirds suggest convergent regulatory mechanisms underlying auditory perception.

## Introduction

Playing an instrument requires meticulous integration between the sensory (visual, auditory, tactile) and motor systems (timing, sequencing and spatial organisation of movements) (Zatorre, Chen & Penhune, 2007; Hyde et al., 2009). Further, orchestral performance requires synchronisation between fellow musicians and the conductor via non-verbal visuomotor and audiomotor communication, underscoring enhanced cooperation between musicians (Volpe et al., 2016). Long-term focused sensorimotor training often required for mastering a musical instrument causes structural and functional changes in the brain and cerebral cognitive networks (Schlaug, 2001; Gaser & Schlaug, 2003; Meyer, Elmer & Jäncke, 2012; Herholz & Zatorre, 2012; Moore et al., 2014). Accordingly, neural processing of music components and activation of different brain areas differ significantly between musicians and non-musicians (Park et al., 2014).

Research based on the effect of music training on the musician’s brain has often used neuroimaging techniques and descriptive analyses (Elbert et al., 1995; Schlaug, 2001; Gaser & Schlaug, 2003; Hyde et al., 2009; Meyer, Elmer & Jäncke, 2012; Herholz & Zatorre, 2012; Steele et al., 2013; Moore et al., 2014; Park et al., 2014). By applying genomic approaches, we and others have previously reported genetic predisposition in shaping human musical aptitude (Pulli et al., 2008; Park et al., 2012; Ukkola-Vuoti et al., 2013; Oikkonen et al., 2015). Music can also cause epigenetic alterations in the human body: we have shown that music-performance and music-listening affect expression of several genes, many of which are regulated by singing in songbirds (Kanduri et al., 2015a, 2015b). Indeed, songbirds offer important models for studying genomic mechanisms underlying sound perception and production (vocal communication) and social communication in humans (Whitney et al., 2014).

MicroRNAs are small non-coding RNAs of approximately 18–22 bp in length that can post-transcriptionally regulate approximately 60% of human genes, including those that play a critical role in neuronal functions like synaptic plasticity (Huntzinger & Izaurralde, 2011; Olde Loohuis et al., 2012; Wang, Kwon & Tsai, 2012). Further, studies on synaptic dynamics indicate that synaptic efficiency and size can be modified in a time frame of minutes (Regehr, 2012; Altenmüller & Schlaug, 2015). Interestingly, studies on songbirds indicate regulation of microRNAs in the auditory forebrain with functional implications on neuron differentiation and neuronal plasticity (Gunaratne et al., 2011). Here, we studied changes in the peripheral blood microRNA transcriptome after 2 hours of concert performance in professional musicians using high-throughput sequencing and computational methods. We have used peripheral whole blood as functional human brain tissues are inaccessible.

## Materials and methods

### Ethical permission

The study was conducted in accordance with the guidelines of Helsinki Declaration and ethical permission (number 233/13/03/2013) has been approved by the Ethical Committee of Helsinki University Central Hospital. Additionally, a written informed consent has been obtained from the participants.

### Music-performance

The music-performance study was comprised of professional musicians (*N* = 10) from *Tapiola sinfonietta* who performed a Western classical music concert (duration approximately 2 hours) to the public (see Supplemental Methods). Seven of the 10 participants were also participants of our previous gene expression study (Kanduri et al., 2015a). The program for the concert comprised five music pieces described earlier (Kanduri et al., 2015a). We then collected verbal data from all the musicians regarding their activities before the concert (coffee/alcohol consumption), personal ratings (pleasantness of the concert and blood sample collections, conductor’s influence on the music-performance, mood of the musicians before and after the concert, fondness for the played music pieces and familiarity of the pieces played) and stress factors for the day before concert and for the concert day using a questionnaire, described in detail in Kanduri et al. (2015a).

### Control study

The control group consisted of professional musicians (*N* = 10); but they neither played music nor listened to music during the control study (see Supplemental Methods). Instead, the musicians could choose either to attend a lecture or walk outside for 2 hours. The characteristics of the control group of professional musicians (*N* = 10) has been described earlier (Kanduri et al., 2015a). Using the same participants and music exposure as in the gene expression study (Kanduri et al., 2015a) enables to integrate the findings to understand the microRNA:mRNA interaction and the regulatory network in professional music-performance.

### Sample collection, RNA extraction and sequencing

Peripheral whole blood samples (2.5 ml) were collected from the musicians immediately before and immediately after the music-performance and control study. The details of sample collection, microRNA extraction and sequencing are provided in the Supplemental Methods.

### Pre-processing and statistical analysis of microRNA expression

The reads from the microRNA sequencing runs were quality controlled with FastQC version 11.3 (http://www.bioinformatics.babraham.ac.uk/projects/fastqc/) and trimmed to remove the 3′ adapter sequence and low quality reads using Trim Galore! Version 0.3.7 (http://www.bioinformatics.babraham.ac.uk/projects/trim_galore/). An adapter overlap of five bp, read length threshold of 15 bp, Phred score quality cut-off of 20 and an error rate threshold of 0.1 were applied for adapter and quality trimming. The reads were quality checked again using FastQC and aligned to the human genome reference (GRCh38, Ensembl release 76) using bowtie version 1.1.2 (Langmead et al., 2009) requiring perfect match for a seed length of 18 and selecting only unique alignments. The microRNA expressions were calculated from the alignments using HTSeq release 0.6.1p1 (Anders, Pyl & Huber, 2015) with annotations from the miRBase version 21 (Kozomara & Griffiths-Jones, 2014).

For the statistical analysis of microRNA expression differences over time (before vs. after 2 hours) across the music-performance and the control study, we used DESeq2 (version 1.22.1) (Love, Huber & Anders, 2014). DESeq2 performs very conservative testing based on negative binomial distribution of the sequencing counts with optimal balance between specificity and sensitivity (Khang & Lau, 2015; Tang et al., 2015) by: (1) estimating normalisation factors to account for sequencing depth differences between samples, (2) calculating microRNA-wise dispersions, (3) fitting negative binomial generalised linear model (GLM) and (4) by performing statistical significance testing. For this study, we used a GLM with a design formula containing the condition factor (music-performance and control activity (reference)), the time factor (Post and Pre (reference)), their interaction and the paired experimental design (R code: *dds <- DESeqDataSet(dds, ∼ Pair + treatment + time + treatment:time*)). We then performed the likelihood ratio test with a reduced model which does not contain the interaction term to find microRNAs that changed their expression over time (2 hours), as a result of music-performance, compared to the control activity (music-performance vs. control), as suggested in DESeq2 vignette (R code: *dds <- DESeq(dds, test=‘LRT’, reduced = ∼ Pair + treatment+ time*)). Additionally, to determine microRNAs with significant differential expression, we applied the Benjamini–Hochberg’s false discovery rate (FDR) threshold of 5%. Further, we selected only those differentially expressed microRNAs with a fold threshold of at least 1.3 over time (post vs. pre) across conditions (music-performance vs. controls), and post–pre threshold change of at least 15% for the music-performance study as candidate microRNAs.

Some important aspects of assigning fold change thresholds to individual studies should be taken into consideration here. The choice of thresholds to prioritise the top candidates from a study is based on the study question and the number of features (genes or microRNAs) identified as differentially expressed (Wu, Wang & Wu, 2015). Furthermore, our study represents gene–environment interaction where lower expression fold changes has been observed (Radom-Aizik et al., 2012, 2014; Black et al., 2013; Beech et al., 2014; Osterndorff-Kahanek et al., 2018).

### Functional over-representation analysis of microRNAs

We assessed the functions of the differentially expressed (DE) microRNAs using two microRNA specific enrichment tools, TAM 2.0 (Li et al., 2018) and miRNA enrichment analysis and annotation tool (miEAA) (Backes et al., 2016) due to the bias observed in the functional enrichment analysis of the target genes of microRNAs by standard enrichment tools (Bleazard, Lamb & Griffiths-Jones, 2015). TAM 2.0 performs over-representation analysis (ORA) using hypergeometric test to test whether the input microRNAs are over or under-represented in the reference microRNA annotation sets (associated with functions and diseases) specifically curated for microRNA genes. TAM 2.0 also calculates the correlations between the input set of microRNAs and microRNAs which were found to be deregulated in other disease conditions based on literature findings. Whereas, miEAA performs ORA at the level of mature microRNAs for categories (ontology, pathway or diseases) and tests whether a category is significantly enriched (Fisher’s exact test) in a given microRNA set with respect to the reference using statistical test implemented in the gene set analysis toolkit, GeneTrail. It has to be noted here that majority of functions for the microRNAs in the reference annotation set used in miEAA for the gene ontology ORA are derived mainly based on the functions of their target genes.

Using TAM 2.0, we performed ORA of functions and diseases for our up-regulated and down-regulated microRNAs separately with default options (FDR <0.05) and with cancer terms masked. We then used miEAA for ORA of our up-regulated and down-regulated microRNAs separately with default options (FDR <0.05) selecting all the categories and used all annotated microRNAs/precursors collected in miEAA as reference set. To relate the microRNA expression patterns in blood to that of brain, we verified tissue specificity of the DE microRNAs and their target genes using microRNA databases, other databases (BBBomics) (Kalari et al., 2016) and literature searches.

### MicroRNA target gene detection and their functional relevance

To understand microRNA regulatory mechanisms and networks in music-performance, we collected the validated target gene interactions for each DE microRNA with strong evidence (reporter assay or western blot) from miRTarBase Release 7.0 (Chou et al., 2018). Additionally, predicted target genes for each of the DE microRNAs were gathered from TargetScan algorithm Release 7.2 (Agarwal et al., 2015) using the below filtering criteria to reduce the false positive target genes. For the conserved and broadly conserved microRNA families, we chose only the target genes with conserved sites, having an aggregate probability of conserved targeting at least 0.2 and a total context++ score at most −0.15. For the poorly conserved DE microRNA families and those with other miRBase annotations, target genes with a total context++ score of less than −0.15 were selected (Agarwal et al., 2015). We did not consider predicted target genes of the DE microRNAs with non-canonical binding. Next, we extracted the latest validated ontology annotations derived using experimentally verified miRNA:target interaction data, named *EBI-GOA-miRNA* (Huntley et al., 2018), via the PSICQUIC web service. Additionally, we also searched the Rat Genome Database (Shimoyama et al., 2015) for annotations of our conserved microRNA orthologs, as per the Alliance of Genome Resources, from *Rattus norvegicus*.

### Comparative analyses

We assessed conservativeness of the microRNA regulatory mechanisms associated with music perception and production in professional musicians by comparative analysis to the microRNA regulatory patterns identified in the songbird brain after song-listening (Gunaratne et al., 2011) and singing (Shi et al., 2013). We then compared target genes of the differentially regulated microRNAs after music-performance to the target genes of song-listening and singing responsive microRNAs from songbirds (Gunaratne et al., 2011; Shi et al., 2013; Lin, Balakrishnan & Clayton, 2014). Next, we tested the overlap between the target genes of the down-regulated microRNAs from our study and the genes stimulated by singing in songbirds. Towards this, we compiled a dataset comprising genes which were previously identified to show behavioural regulation (up-regulation) after singing in songbird brain based on findings from three studies (Wada et al., 2006; Hilliard et al., 2012; Whitney et al., 2014) and call it *song production cum perception gene set*. We then implemented a permutation test to determine whether the observed overlap between the *song production cum perception gene set* and the target genes of the down-regulated microRNAs from our study was more than that expected by chance through re-sampling (*N* = 10,000 times) of the datasets. Specifically, we randomly sampled (without replacement) the songbird genes of the same size as *song production cum perception gene set* using all the annotated genes from *Taeniopygia guttata* (*N* = 17,926) as Universe. Similarly, human genes were randomly sampled (without replacement) for the same size as the number of predicted and validated target genes of the down-regulated microRNAs using annotated human genes as the Universe (*N* = 20,219). We next estimated the overlap between these re-sampled datasets for each of the permutations of these datasets and calculated the *p*-value of the overlap. Similarly, we also analysed the statistical significance of the overlap between target genes of the up-regulated microRNAs from our study and the genes found inhibited after singing in songbird brain (Whitney et al., 2014) using re-sampling (*N* = 10,000).

### Integrated analysis and putative regulatory network construction

Our objective with the integrated analysis of our microRNA results with the gene expression data in professional music-performance (Kanduri et al., 2015a) was to create a putative gene regulatory network that represents the molecular mechanisms underlying music-performance in professional musicians. We created microRNA–gene regulatory interactions using the target genes collected from miRTarBase, TargetScan and using Qiagen’s Ingenuity® Pathway Analysis (IPA®; Qiagen, Redwood City, CA, USA, www.qiagen.com/ingenuity). Only those target genes of the differentially expressed microRNAs from this study which showed inverse direction of regulation in the gene expression findings, from the same music-performance and control activity as this study (Kanduri et al., 2015a), were considered for generating microRNA–gene regulatory interactions.

Additionally, for constructing the putative regulatory network, we have considered microRNA-transcriptional factor (TF), TF-genes and TF-microRNA regulatory information. We considered microRNA-TF regulatory data as microRNAs, which can be under the regulation of transcriptional regulators, can regulate genes either directly or indirectly through the regulation of transcription factors (Otaegi et al., 2011; Li et al., 2017). The transcriptional regulatory data for the DE genes (TF-gene) were obtained from Table S4 of the same study (Kanduri et al., 2015a), which comprises the statistically significant upstream regulators for the DE genes. Additionally, for this study, we collected the regulatory effect (activation/inhibition) of the up-stream regulator on the target gene and their validation status for incorporating the information to the network. MicroRNA–TF interaction data were gathered from TargetScan, miRTarBase and literature for identifying the interaction between DE microRNAs and the over-represented transcriptional regulators of the DE genes (Kanduri et al., 2015a) after considering their putative regulatory effect on the DE genes. Validated TF-microRNA regulatory data were collected from TransmiR-2 (Wang et al., 2010) and literature search. Further, only those TFs previously found to be associated with musical traits (Oikkonen et al., 2016) and/or found in songbird singing or listening (Wada et al., 2006; Hilliard et al., 2012; Whitney et al., 2014) were considered for the putative regulatory network construction. This putative regulatory network was further extended with validated target genes of the DE microRNAs that were found both from miRTarBase and *EBI-GOA-miRNA*, their putative neighbours, co-expressions and indirect regulators gathered using the STRING database (Szklarczyk et al., 2017) and extensive literature search to connect the microRNA and gene expression findings and visualised with Cytoscape 3.7.0 (Shannon et al., 2003).

## Results

### Comparison of the music-performance and control study participants

We chose professional performing musicians as they start their music training during sensitive period of human brain development (approximately 6 year of age) (Steele et al., 2013), undergo rigorous training and constantly practice to maintain their expertise (Pallesen et al., 2010; Oechslin et al., 2013). Table 1 shows detailed demographic information for the professional performers (*N* = 10) and control musicians (*N* = 10) and their statistical comparison.

**Table 1:** Phenotypic characteristics of the participants.

Characteristic	Music-performance (*N* = 10)	Control study (*N* = 10)
Female	6	8
Age	48.3 ± 6.17; CI = 4.41	39.2 ± 11.31; CI = 8.09
Age at commencing of music practice	6.6 ± 1.71; CI = 1.23	5.7 ± 1.64; CI = 1.17
Average hours of practise/day	5.3 ± 0.82; CI = 0.59	4.3 ± 2.06; CI = 1.47
Keyboard musicians	0	3
String musicians	10	4
Wind musicians	0	3
University degree (B.Mus./Diploma/Master’s)	10	10

**Note:**

Participant demographic data are furnished in this table. Continuous explanatory variables—age, age at commencing of music practice and average hours of practice, are presented as the mean ± standard deviation with confidence interval (CI) at 95%. To assess the similarity of the music-performance and control participants, we performed two-sided *t*-tests to compare the age of commencement of musical practice, age of the participants and number of hours of musical practice per day. At 95% confidence, we did not find any significant (*p* < 0.05) differences in the age of commencement of musical practice, age of the participants and the number of hours of musical practice per day between music-performance and control groups. Additionally, we performed two-sided fisher’s exact test for count data to assess any statistically significant differences between the gender and music education level of the music-performance and control groups. However, at 95% confidence, we did not find any statistically significant differences in the gender of the participants between music-performance and control groups. Similarly, the education level of the performing musicians and the control group did not show any statistically significant differences from each other.

### MicroRNA response to music-performance

**Figure 1:**
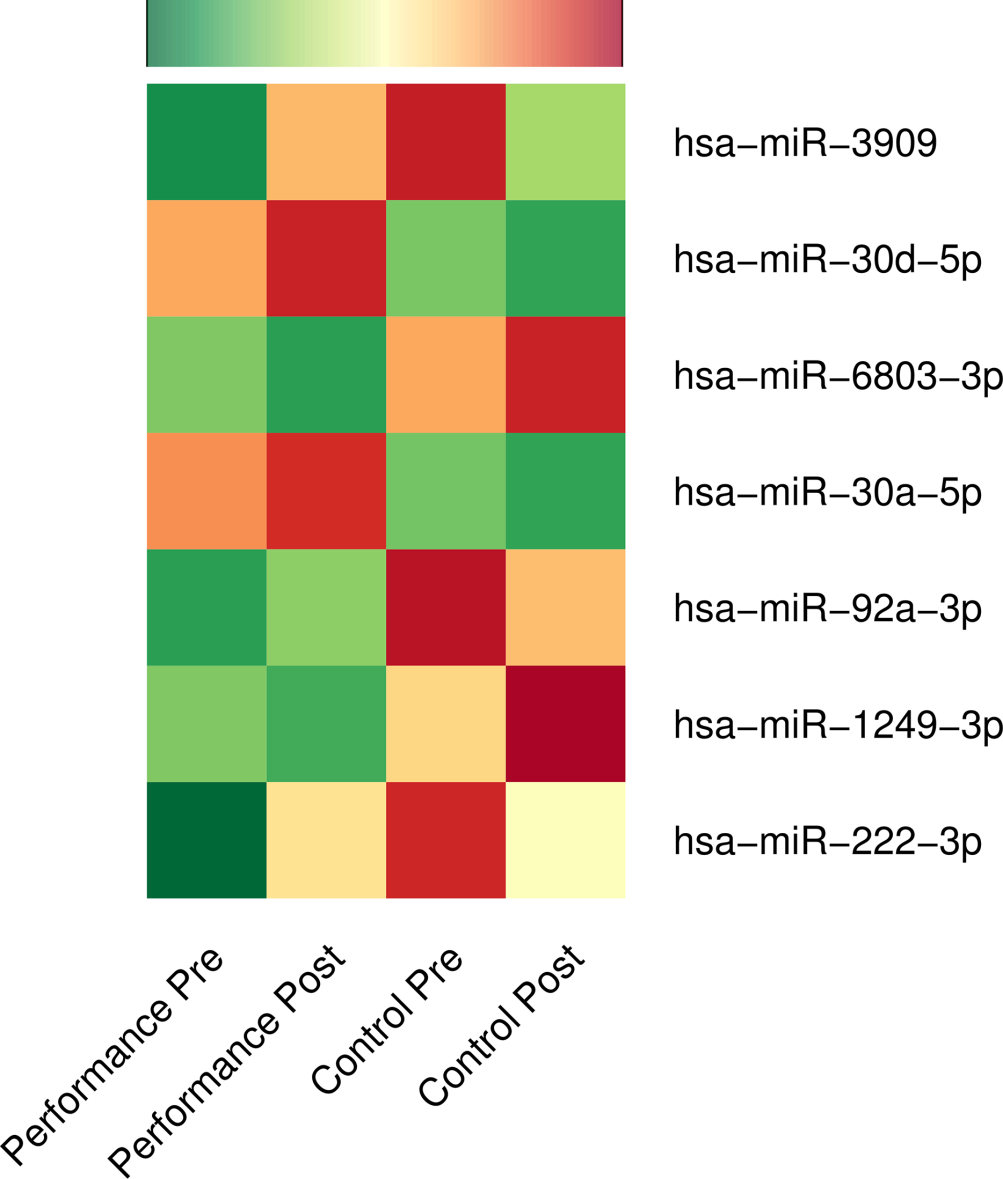
Differential expression of microRNAs: music-performance vs. controls. Normalised mean expressions of differentially regulated microRNAs before and after music-performance/control studies are represented using heatmap. Green, yellow and red colours denote low, moderate and high expression of microRNAs, respectively.

We compared the microRNA expressions over time (post vs. pre) across the music-performance and the control activity with DESeq2 (Love, Huber & Anders, 2014) owing to its strengths mentioned below. DESeq2 is one of the most sensitive statistical tools for performing differential expression analysis of high throughput sequencing experiments with a wide range of sample numbers as well as for those with a smaller fold changes (Love, Huber & Anders, 2014; Zhou, Lindsay & Robinson, 2014). Furthermore, systematic comparison of statistical tools for analysing differential expression shows DESeq2 giving a false positive rate as low as 0 and true positive rate above 80%, even with a log fold threshold as low as 0.5 and with a replicate number of 6 (Schurch et al., 2016), making DESeq2 a statistically appropriate tool for this study. At a stringent FDR threshold of 5%, we identified significant up-regulation of five microRNAs (hsa-miR-3909, hsa-miR-30d-5p, hsa-miR-92a-3p, hsa-miR-30a-5p, hsa-miR-222-3p) and down-regulation of two microRNAs (hsa-miR-6803-3p and hsa-miR-1249-3p) over time (2 hours) after music-performance, compared to the control activity (Fig. 1; Table S1). Genomic information for the DE microRNAs is provided in Table S2 and a concise list of putative functions of the up-regulated microRNAs and selected target genes of the down-regulated microRNAs in Table 2. The up-regulated microRNA gene hsa-mir-30d is located on 8q24.22, near to 8q24, which has been implicated in absolute pitch (Theusch, Basu & Gitschier, 2009) and auditory neuropathy (Rance, 2005). Likewise, hsa-mir-3909 is located on 22q12.3, whose deletions (22q12.3–q13.1) are associated with neurocristopathy and sensorineural hearing loss (Jelena et al., 2014).

**Table 2:** Putative functions of the differentially regulated microRNAs and their target genes in music-performance.

Up-regulated microRNAs	Putative functions
hsa-miR-30d-5p	Responsive to song-listening in songbird forebrain. Takes part in auditory, sensory, nervous system development and functioning, myelination, vascular remodelling.
hsa-miR-222-3p	Responsive to song-listening in songbird forebrain. Takes part in nervous system development, inner ear development, neuron differentiation, neuronal plasticity, maintenance of long-term potentiation, Schwann cell regeneration, sensory perception of sound, angiogenesis, cardiac growth, negative regulation of apoptosis.
hsa-miR-92a-3p	Motor neuron differentiation, synaptic plasticity, long-term synaptic potentiation, sensory perception of sound, negative regulation of apoptosis, regulation of heart contraction
hsa-miR-30a-5p	Schwann cell differentiation, neuron differentiation, myelination, negative regulation of apoptosis, vascular remodelling.
hsa-miR-3909	Neuroprotection

Target genes of up-regulated microRNAs	Putative functions
*PTEN, CASP3*	Apoptosis
*FOXP2*	Song perception and production, sensorimotor learning, vocal plasticity
*CDKN1B*	Cell-cycle inhibition
*IL-1, IL-6, IL-8*	Pro-inflammatory cytokines

**Note:**

Functions of up-regulated microRNAs and their targets were gathered from literature. Down-regulated microRNAs are not well-characterised for functions or target genes.

### Putative functions of the DE microRNAs

From the analysis of the DE microRNAs using TAM 2.0, the up-regulated microRNAs were found to have functions in aging, chondrocyte development, angiogenesis, regulation of Akt pathway and cell death (Data S1). The DE microRNAs, hsa-miR-222-3p and hsa-miR-92a-3p activates AKT (AKT serine/threonine kinase 1) signalling through their targeting of *PTEN*, which is an activator of apoptosis and inhibitor of AKT. Akt is activated by growth factors, insulin (Hayashi et al., 2017) and is regulated through phosphatidylinositol 3-OH kinase (PI3′K) signalling, implicated in dopamine neurotransmission (Tan et al., 2008) and promotes cell survival, cellular proliferation and growth signalling, in part, through the activation of the transcriptional regulator, *CREB* (Du & Montminy, 1998). According to Entrez gene annotation, activated AKT suppress apoptosis and is involved in the growth factor-mediated neuronal survival in the developing nervous system. This is consistent with microRNA findings from songbird learning (Gunaratne et al., 2011) where the target genes of the down-regulated microRNA activated MAPK signalling pathway, another pathway with roles in cellular proliferation and neuronal plasticity, and confirms some of the findings from human music-performance (Kanduri et al., 2015a) and music-perception (Kanduri et al., 2015b). Interestingly, conditional inactivation of AKT1 has been linked to the hyperphosphorylation of TAU protein which is implicated in Alzheimer’s disease (Wang et al., 2015). The up-regulated microRNAs repressed cell death and were found to be interfering with damaging cues.

Based on the analysis of the microRNAs using miEAA, the up-regulated microRNAs were found to be regulators of apoptotic processes implying pro-survival effects associated with music-performance. Other functions regulated by the up-regulated microRNAs include sensory perception, peripheral nervous system axon regeneration, intracellular transport, cytokine production and primary microRNA processing (Data S2). Furthermore, miR-30d and miR-222 in *R. norvegicus* are involved in long-term synaptic potentiation and sensory perception of sound (Smith et al., 2018). The functions and tissue expression data of the candidate microRNAs based on annotations from microRNA databases and from extensive literature analysis are given in Table S3.

### Target genes and comparative analyses with genetic studies on song perception and production

**Figure 2:**
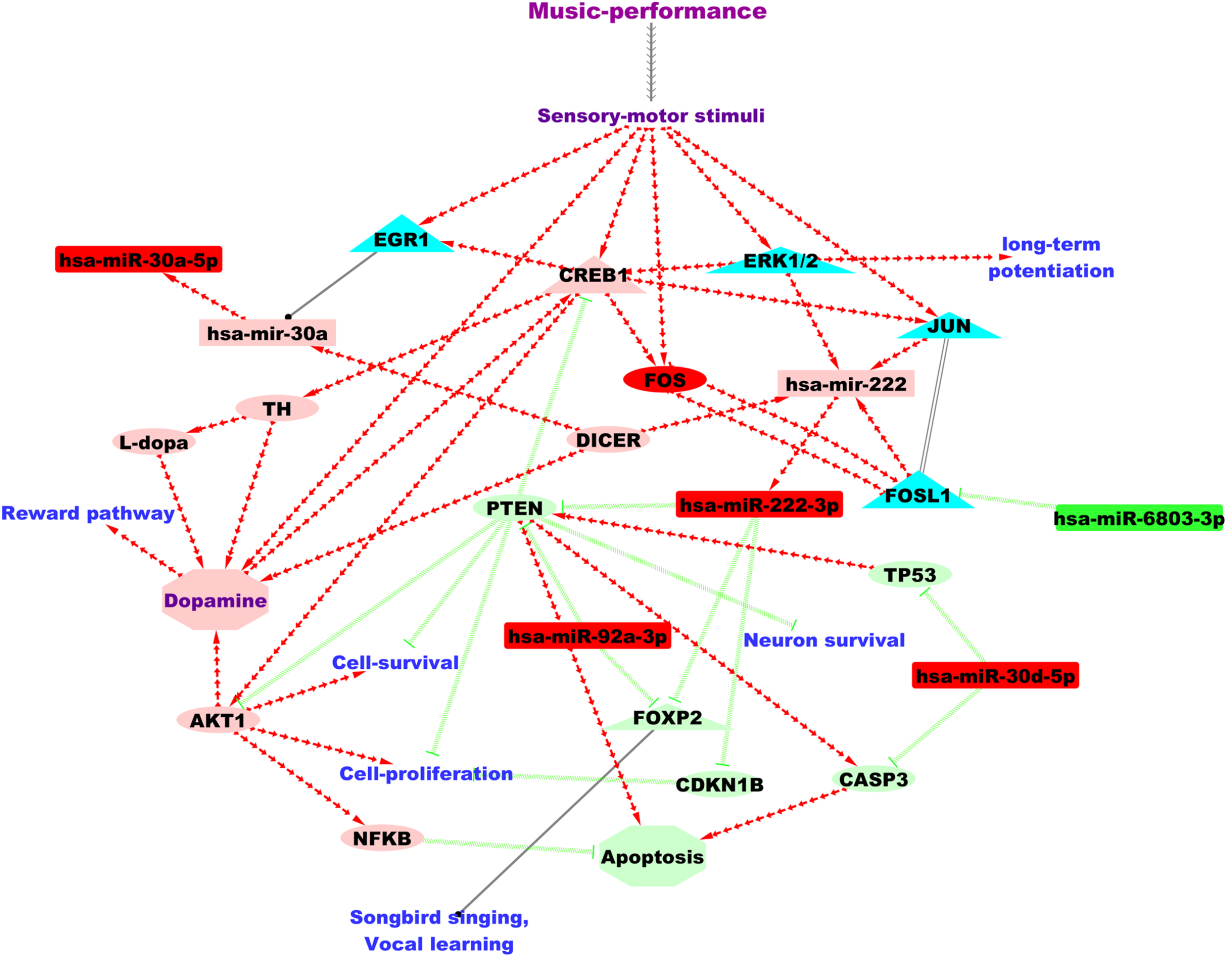
A putative microRNA regulatory network and summary of the findings from the current study based on the analysis of the DE microRNAs. Green and red colour nodes, respectively, indicate down-regulated and up-regulated microRNAs from our study. Light-green and coral colour nodes denote putative repression and activation, respectively, based on findings from microRNA databases and literature. Annotation for edges: Red separate arrow edges denote activation; green backward slashes indicate repression; solid black edge denotes regulation and parallel lines indicate co-expression. MicroRNAs are represented using rectangles, genes using ellipses and transcriptional regulators using triangles.

Altogether, we collected 2,027 and 3,825 target genes (validated and predicted inclusive), respectively, for the down-regulated and up-regulated microRNAs from both TargetScan and miRTarBase. Out of these, validated human target genes supported by strong experimental evidence were observed only for the up-regulated microRNAs (*N* = 143) from miRTarBase. Validated target genes and GO annotations as per *EBI-GOA-miRNA* dataset (Huntley et al., 2018) were only available for three of the seven DE microRNAs: hsa-miR-30a-5p, hsa-miR-222-3p and hsa-miR-92a-3p. Based on *EBI-GOA-miRNA* dataset, hsa-miR-92a-3p and hsa-miR-222-3p were involved in the negative regulation of inflammatory response (GO:0050728) and angiogenesis (GO:0016525). Additionally, we identified *PTEN*, a promoter of apoptotic processes, as the validated target shared by hsa-miR-222-3p and hsa-miR-92a-3p from *EBI-GOA-miRNA* dataset. A putative microRNA regulatory network underlying professional music-performance based on findings from the current study and its functional relevance is summarised in Fig. 2.

Comparative analyses to songbirds identified three of the up-regulated microRNAs from this study (miR-30, miR-222 and miR-92) as regulated in the auditory forebrain of songbirds after song-learning (Gunaratne et al., 2011). Further, two of the microRNAs found up-regulated after music-performance in the professional musicians in our study, hsa-miR-92a-3p and hsa-miR-222-3p, target *FOXP2* and constitute a microRNA-*FOXP2* gene regulatory network. This is in accordance with findings from songbirds in which undirected singing behaviour up-regulated two microRNAs in the brain (miR-9 and miR-140-5p) that were regulators of the *FOXP2* gene, which is important for vocal learning (Shi et al., 2013). Amongst the predicted target genes of the song-learning (listening) inhibited miR-2954 (Gunaratne et al., 2011), we identified *TLK2* as putatively regulated by hsa-miR-6803-3p, which is down-regulated after music-performance. hsa-miR-6803-3p is also predicted to target *PRKCA*, which was up-regulated during song-responsive down-regulation of miR-2954 (Lin, Balakrishnan & Clayton, 2014). Similarly, we also observed putative targeting of *RPS11* and *RPL29*, which were down-regulated after miR-2954 inhibition (Lin, Balakrishnan & Clayton, 2014), by hsa-miR-3909, one of the up-regulated microRNAs from this study.

From the analysis of the significance of the gene-set overlap based on permutation test, the overlap observed between target genes of the down-regulated microRNAs and the genes stimulated by singing in songbirds, *song production cum perception gene set*, is more than that expected by chance (*p*-value = 0.0017). Likewise, we also identified a statistically significant overlap between the genes down-regulated after songbird singing (Whitney et al., 2014) and the target genes of the up-regulated microRNAs (re-sampling *p*-value = 0, *N* = 10,000); 33.33% of the genes down-regulated after songbird singing (Whitney et al., 2014) were amongst target genes of the up-regulated microRNAs (Table S4).

## Integrated analyses of music-performance responsive micrornas and genes

Differentially expressed microRNAs from this study were expression paired and assessed for inverse regulatory patterns with the RNA expression study, which studied the same music-performance in the same musicians (Kanduri et al., 2015a), with IPA and using the collected target genes. From this integrated analysis, we observed predicted interaction of the down-regulated hsa-miR-1249-3p with one of the up-regulated genes, *OSBP2*, from the gene expression analysis (Kanduri et al., 2015a) (Table 3). Likewise, we identified predicted interactions of the up-regulated microRNAs with down-regulated genes (Kanduri et al., 2015a); hsa-miR-222-3p with *NBPF9*; hsa-miR-3909 with *NBPF9*, *CNPY3*, *CYTH4*, *ABI3*, *ADRB2*; hsa-miR-30d-5p and hsa-miR-30a-5p with the down-regulated *CHD9* and *ADRB2* (Table 3). We also observed the differentially regulated microRNAs from our study to have predicted interactions with statistically over-represented upstream regulators of genes differentially regulated in professional music-performance (Table 3).

**Table 3:** MicroRNA:gene and microRNA:TF interactions (direct) associated with 2 hours of professional music-performance obtained by integrating microRNA expression profiles, gene expression profiles and statistically significant up-stream regulators of the DE genes.

Comparison	Target genes for the DE microRNAs from the differentially regulated genes
Up-regulated microRNA interactions with down-regulated genes from (Kanduri et al., 2015a)	*NBPF9, CNPY3, CHD9, CYTH4, ABI3, ADRB2*
Down-regulated microRNA interactions with up-regulated genes from (Kanduri et al., 2015a)	*OSBP2*
**Up-regulated microRNA**	**Validated and predicted interactions with upstream regulators of down-regulated genes from gene expression study (Kanduri et al., 2015a)**
hsa-miR-222-3p	FOXP2
hsa-miR-92a-3p	FOXP2
hsa-miR-30a-5p and hsa-miR-30d-5p	NHLH2
**Down-regulated microRNA**	**Predicted interactions with upstream regulators of up-regulated genes from gene expression study (Kanduri et al., 2015a)**
hsa-miR-6803-3p	FOSL1

## Putative microrna regulatory network in professional music-performance

**Figure 3:**
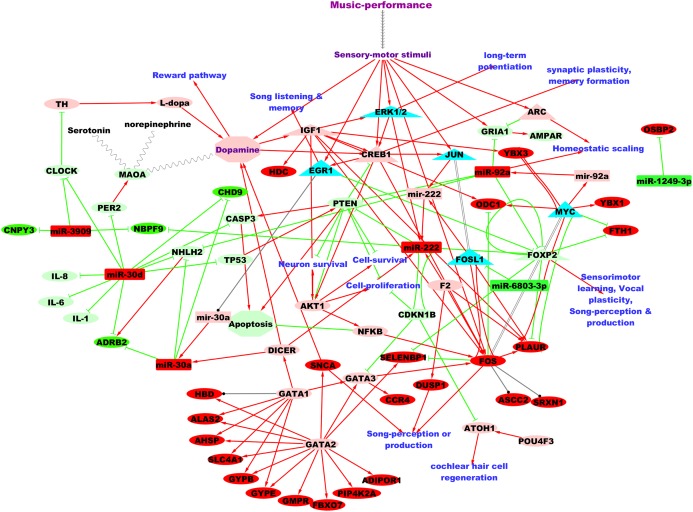
Putative TF:microRNA:gene regulatory network in professional music-performance. Candidate microRNAs are denoted by rectangles, target or other genes by ellipses and transcriptional regulators of candidate microRNAs by triangles. Green and red colour indicates down-regulation and up-regulation of the molecules (microRNAs and genes), respectively, after professional music-performance. Light-green and light-red colour nodes denote putative repression and activation, respectively, based on findings from microRNA databases and literature given in Discussion. Red arrows, green edges and black edges, respectively, show putative activation, repression and regulation. Solid and dotted arrows show direct and indirect interactions, respectively.

Figure 3 shows the putative microRNA regulatory network underlying 2 hours of professional music-performance generated from the integrated analysis with the RNA expression study (Kanduri et al., 2015a), incorporated with relevant findings from microRNA databases and the literature findings. Furthermore, the functional interactions between the genes which were up-regulated after music-performance (Kanduri et al., 2015a) from the integrated analysis (Fig. 3) and the putatively activated molecules from Fig. 3 (coral colour ellipses) were deduced using STRING and is given in Fig. S1. Interestingly, some of the molecules in the network were important for cell survival, cellular proliferation, neuron survival, long-term potentiation and dopamine signalling. Some of the molecules such as *GATA2*, *GATA3* and miR-222 have functions in the inner ear development. Selected validated target genes from amongst this putative regulatory network (Fig. 3) are mentioned in detail in the Discussion.

## Discussion

Here, we report differential regulation of peripheral blood microRNAs in professional musicians post 2 hours of Western classical concert performance relative to a non-music control activity of the same duration using high-throughput sequencing and computational methods. Majority of the DE microRNAs were up-regulated, in accordance with microRNA regulation observed after singing in songbirds (Shi et al., 2013). The up-regulated microRNAs were found to repress apoptotic processes and inflammatory cytokines; activate (indirectly) the AKT signalling pathway and its transcriptional target, *CREB* (Du & Montminy, 1998), which are implicated in dopamine neurotransmission (Tan et al., 2008), cell survival (neuron), cellular proliferation and neuronal plasticity. These findings are consistent with the endogenous dopamine release observed during music-listening (Salimpoor et al., 2011) and the changes in brain plasticity identified in connection with professional music-training (Schlaug, 2001; Gaser & Schlaug, 2003; Groussard et al., 2010; Pantev & Herholz, 2011; Herholz & Zatorre, 2012). Here, we acknowledge that peripheral blood was used to study and interpret the changes in brain. This is facilitated by the highly similar (>80%) transcriptome shared by human peripheral whole blood and brain (Liew et al., 2006; Liang et al., 2007) and the positive correlation observed between peripheral blood and auditory forebrain transcriptomes (>70%) after song-listening in songbirds (Louder, Hauber & Balakrishnan, 2018). It is also plausible that environmental stimuli that elicit microRNA changes in the brain produce similar changes in the periphery, as illustrated in the brain-to-periphery model by Rao, Benito & Fischer (2013). Additionally, all the up-regulated microRNAs were expressed in the blood-brain barrier (Kalari et al., 2016) and various parts of the nervous system (Table S3), suggesting their brain expressions. In fact, multiple studies on human brain diseases have correlated the blood microRNA expression differences to their profile changes in the brain; for example, Parkinson’s disease (Martins et al., 2011), stroke (Liu et al., 2010) and Alzheimer’s (Leidinger et al., 2013). Accordingly, some of the observed microRNA changes and the regulatory mechanisms (Fig. 3) could reasonably reflect their changes in the brain.

We acknowledge here that factors that are not intrinsic to music such as the degree of emotional arousal of the participants during music-performance (Nakahara et al., 2011), stress level of the participants, familiarity of the music, mood of the musicians, pleasantness of the event etc. may have affected the results. To account for these, we collected some of these information from the music performers using a questionnaire (see Methods). Although the performing musicians reported to be slightly or moderately nervous before the performance, some of the music pieces they performed were very familiar to them and most musicians described their mood before and after the event as pleasant. We also acknowledge that these factors were not empirically studied in this work. Nonetheless, the long experience in performing as members of classical symphony orchestra (*Tapiola sinfonietta*) to the public suggested that the musicians were habituated to their venue of performance, well-versed with performing and had good rapport between their fellow musicians. Notably, all the musicians liked the music pieces they played, rehearsed the music pieces for the concert at the venue of their performance on the morning of the concert day and most musicians reported the whole event as relatively pleasant, supportive of the fact that these factors might have boosted their confidence, valence and quality of performance (Dib & Sturmey, 2011). Further focused studies using different settings are required to assess the effect of each of these different factors on the performing musicians.

Two of the microRNAs up-regulated after music-performance from this study, miR-30d and miR-222, corroborate findings from songbirds, the widely used neuroscientific model, in which they are differentially regulated in the auditory forebrain during song-learning/listening (Gunaratne et al., 2011). DE microRNAs from this study also show predicted interactions with some of the differentially regulated genes which are responsive to song-stimulated inhibition of miR-2954 (Gunaratne et al., 2011; Lin, Balakrishnan & Clayton, 2014). Further, putative target genes of miR-2954 and the up-regulated microRNAs from this study activate pathways and functions that promote cell survival and cellular proliferation related to neuronal plasticity (Gunaratne et al., 2011; Lin, Balakrishnan & Clayton, 2014).

The up-regulated microRNAs, hsa-miR-92a-3p and hsa-miR-222-3p target *FOXP2*, which plays crucial roles in human language or speech development (Mozzi et al., 2016) and song learning and singing in songbirds (Haesler et al., 2004; Teramitsu & White, 2006). In agreement with this, upstream regulators (microRNAs and EGR1) bind with FOXP2 promoter in singing songbirds, regulating the corticostriatal FOXP2 (Shi et al., 2013) for brain structure adaptations towards accurate song imitation (Heston & White, 2015), vocal complexity (Haesler et al., 2004) and stabilisation of mature song (Teramitsu et al., 2010). Further, FOXP2 is predominantly a transcriptional repressor and targets *PLAUR*, *SELENBP1* and *FTH1* (Vernes et al., 2007), which were up-regulated after music-performance (Kanduri et al., 2015a), suggesting indirect activation of FOXP2 target genes by microRNAs after music-performance.

miR-222 has been shown to be induced by ERK cascade (Terasawa et al., 2009), which is crucial for memory formation (Adams & Sweatt, 2002), striatal motor neuronal functions (Hutton et al., 2017) and neuronal plasticity (Kelleher et al., 2004). ERK–MAPK signalling pathway, with the help of cyclic adenosine monophosphate, is also involved in the proliferation of supporting cells of the auditory system (Bell & Oberholtzer, 2010). miR-222 also promotes neurite outgrowth by negatively regulating the neuronal growth inhibitor, *PTEN*, and by activating CREB expression and phosphorylation (Zhou et al., 2012). CREB signalling pathway modulates key processes involved in synaptic plasticity and memory formation and is activated by auditory-motor stimuli induced neuronal excitation (Waltereit & Weller, 2003). Interestingly, PTEN is also targeted by the up-regulated miR-92a (Zhang et al., 2014; Ke et al., 2015) and hippocampal *PTEN* knockdown inhibited neuronal apoptosis and *CASP3* (caspase-3) (Zhu et al., 2006). Further, another candidate up-regulated microRNA, miR-30d also targets *CASP3* and is implicated in auditory functions: miR-30d repression after acoustic over-stimulation caused increased caspase-3 expression and apoptosis induced sensory cell degeneration (Patel et al., 2013).

miR-92 and miR-30a are expressed in the brain and promote neuron differentiation (Sempere et al., 2004). miR-92a also plays a role in synaptic plasticity by targeting *GRIA1*, a glutamate receptor and component of AMPA receptor (AMPAR) that is activated by excitatory neurotransmission (Letellier et al., 2014). An immediate early gene, *ARC*, induced simultaneously by neuronal activity, is a key modulator of synaptic homeostatic scaling of AMPAR receptors and top candidate for musical ability (Oikkonen et al., 2016) located on 8q24, close to the up-regulated miR-30d (location:8q24.22).

miR-30 and miR-3909, up-regulated after music-performance, are also predicted to target *CLOCK*, a repressor of *TH* which converts tyrosine to the dopamine precursor, L-dopa (Parekh & McClung, 2015). miR-30 also targets, PER2, another CLOCK protein expressed in the striatum, which positively regulates *MAOA* expression in the mesolimbic dopaminergic system (Hampp et al., 2008; Parekh & McClung, 2015). MAOA is a critical enzyme that metabolises monoamines including serotonin, norepinephrine and dopamine and shows elevated expression in major depression disorders (Meyer et al., 2006). These findings are in accordance with the previous findings of activation of *SNCA* (important for neuron homeostasis, dopamine transmission, song perception of songbirds; Kimpo & Doupe, 1997; Wada et al., 2006) and dopaminergic pathway in connection with music/song perception and production (performance) in humans and in songbirds (zebra finch) (Whitney et al., 2014; Kanduri et al., 2015a, 2015b).

miR-222 is transcriptionally activated by *FOSL1* (Wang et al., 2010), a member of the FOS family of transcription factors, which is co-expressed with *FOS* (up-regulated after music-performance), one of the most likely candidates associated with musical traits (Oikkonen et al., 2016). Also, *FOSL1* and *FOS* are immediate early genes activated by neuronal and acoustic stimulation (Morgan & Curran, 1991) and *FOSL1* is predicted to be regulated by hsa-miR-6803-3p. miR-222 also targets *CDKN1B*, the auditory hair cell cycle inhibitor (Walters et al., 2014; Chou et al., 2018), which encodes p27 protein with a direct repressive effect on *GATA3*, and indirectly activates *GATA3* (Walters et al., 2017). Interestingly, *GATA2* is located on the best associated region for musical aptitude (Oikkonen et al., 2015), transcriptionally activates *GATA3* (Haugas et al., 2016) and *SNCA* co-expression network (Kanduri et al., 2015a, 2015b) and is essential for inner ear development (Lilleväli et al., 2004). Hence, putative repression of *CDKN1B* via miR-222 regulation might be important for the proper functioning of the auditory system (Walters et al., 2017).

Three of the up-regulated microRNAs in this study with a role in vascular remodelling (miR-222, miR-30d and miR-30a), were up-regulated after exercise (Baggish et al., 2011; McLean et al., 2015). This might reflect increased circulation because of the fine-tuning demanding physical activity and accurate motor functions while performing music by professional musicians. miR-30d-5p down-regulation and its inverse correlation between the levels of pro-inflammatory cytokines (IL-1, IL-6, IL-8 and others) were noted in severe sepsis (Caserta et al., 2016) suggesting that its up-regulation associated with music-performance might confer antagonistic effects consistent with findings from the RNA expression study (Kanduri et al., 2015a).

Here, we highlight some of the characteristics shared by professional performing musicians. Irrespective of their speciality, professional performing musicians usually start their music training at a very early age, undergoes rigorous training, constantly practice to maintain their standards excellent and share a multitude of structural and cognitive characteristics (Schlaug, 2001; Gaser & Schlaug, 2003; Meyer, Elmer & Jäncke, 2012; Herholz & Zatorre, 2012; Moore et al., 2014). For example, behavioural research indicate that consistent practice of a cognitive task, like music practice/performance by professional musicians, remodels it from controlled processing to an automated and effortless one with increased speed and accuracy (Jansma et al., 2001). Furthermore, analyses of the social data from musicians provide evidence that factors that might contribute to stress while performing music such as stage fright were found to be lowest in the professional musicians (Steptoe & Fidler, 1987).

## Conclusions

To our knowledge, this is the first global expression profiling of human microRNAs to study professional music-performance at a concert. Our results provide new insights on behavioural microRNA regulatory mechanisms in human music genomics. Taken together, some of the candidate microRNAs and regulatory mechanisms associated with professional music-performance were found in songbird vocalisation and point to evolutionary conservation of sound perception and production. In addition, pathways and biological functions related to neurotransmission and sensorineural plasticity agree with previous studies on music perception and practice. As the genre of music and musical training were found to critically influence perceptual skills and neuronal processing of sound (Vuust et al., 2012), further studies with different genres of music-performance in different study populations are recommended. The impact of gene–environment interactions (including social interactions) on the microRNAs in professional musicians need to be further validated using animal models, preferably using songbirds. Specialised nature of the songbird neural circuits developed through singing experience resembles human music training and the social interaction involved in songbird singing has striking similarities to the non-verbal communication between professional musicians in performance, making songbird an ideal animal model for studying professional music-performance and other music-related human traits.

## Supplemental Information

10.7717/peerj.6660/supp-1 Supplemental Information 1 Significantly over-represented functions for the up-regulated microRNAs based on analysis using TAM 2.0.

10.7717/peerj.6660/supp-2 Supplemental Information 2 Top significantly over-represented biological functions based on gene ontology from the over-representation analysis of the up-regulated microRNAs using miRNA Enrichment Analysis and Annotation tool (miEAA).

10.7717/peerj.6660/supp-3 Supplemental Information 3 Functional interaction between the genes up-regulated after music-performance from the integrated analysis and the putatively activated molecules from Figure 3 as given by STRING. Nodes represent proteins. Edges represent protein–protein functional associations and the colour of the edges represent evidence including co-occurrence (Blue), co-expression (Black), experimental evidence (Purple), fusion evidence (Red), neighbourhood evidence (Green), database evidence (Light blue) and text mining (Yellow).

10.7717/peerj.6660/supp-4 Supplemental Information 4 Tables S1–S4.

10.7717/peerj.6660/supp-5 Supplemental Information 5 Sample collection, RNA extraction and sequencing.

## Additional Information and Declarations

### Data Availability

The following information was supplied regarding data availability: The data reported in this article have been deposited in the Gene Expression Omnibus database under the accession GSE115071.
