# Supplementary figures and images for: Music-performance regulates microRNAs in professional musicians
Source: PeerJ. 2019 Mar 29;7:e6660. doi: 10.7717/peerj.6660 (PMC6442922; doi:10.7717/peerj.6660)

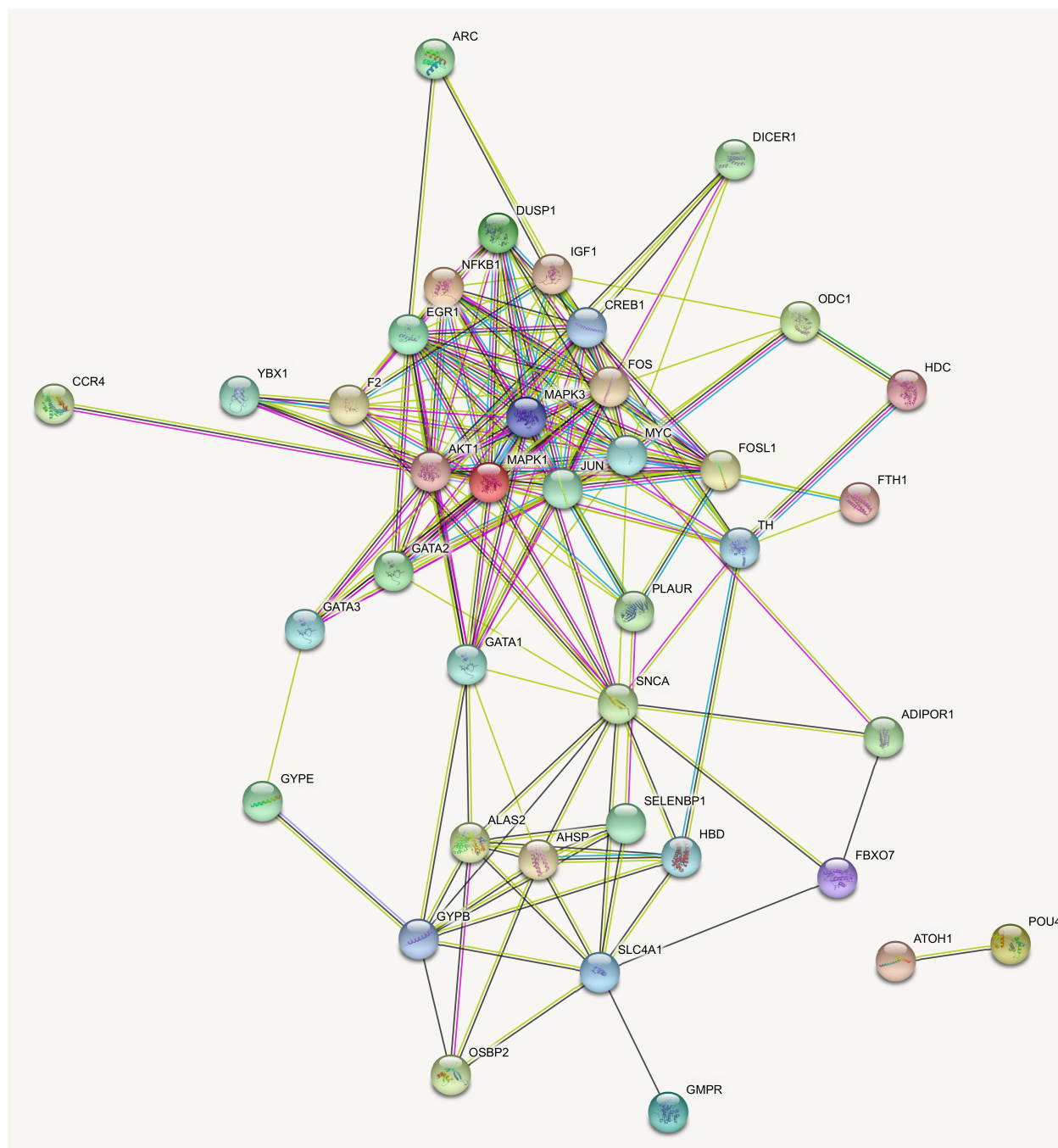

Supplement: Supplemental Information 3 — Nodes represent proteins. Edges represent protein–protein functional associations and the colour of the edges represent evidence including co-occurrence (Blue), co-expression (Black), experimental evidence (Purple), fusion evidence (Red), neighbourhood evidence (Green), database evidence (Light blue) and text mining (Yellow). [file peerj-07-6660-s003.pdf]
