# Supplementary material for: Music-performance regulates microRNAs in professional musicians
Source: PeerJ. 2019 Mar 29;7:e6660. doi: 10.7717/peerj.6660 (PMC6442922; doi:10.7717/peerj.6660)
Supplement: Supplemental Information 4 [file peerj-07-6660-s004.pdf]

Supplemental Tables

microRNA	FC	padj
hsa-miR-3909	1.9443348	0.0002312
hsa-miR-30d-5p	1.5094323	0.0062502
hsa-miR-6803-3p	0.6747345	0.0122642
hsa-miR-30a-5p	1.6681270	0.0206559
hsa-miR-92a-3p	1.3888891	0.0423676
hsa-miR-1249-3p	0.6133912	0.0480158
hsa-miR-222-3p	1.4345961	0.0480158

Table S1. Differentially expressed microRNAs in the music-performance versus control comparison from DESeq2 (Love, Huber & Anders, 2014) analysis. The columns 1-3 show the DE microRNA, fold change and adjusted p-value for music-performance versus control study comparison.

Intragenic DE microRNAs

DE microRNA	Precursor miRNA	Mature miRNAs	Family	Family Conservation	Clustered miRNAs (10kb from the DE microRNA)	Total no. of predicted targets from Targetscan 7.2	Band	Chromosome	Start	End	Host gene
hsa-miR-3909	hsa-mir-3909	hsa-miR-3909	miR-3909	poorly conserved; well annotated	hsa-mir-3909 and hsa-mir-6069	3547 transcripts	22q12.3	chr22	35335640	35335758	TOM1
hsa-miR-6803-3p	hsa-mir-6803	hsa-miR-6803-5p, hsa-miR-6803-3p	miR-6803-3p	other miRBase annotation	hsa-mir-6803 and hsa-mir-6802	1035 transcripts	19q13.42	chr19	55245186	55245250	PPP6R1
hsa-miR-1249-3p	hsa-mir-1249	hsa-miR-1249-3p, hsa-miR-1249-5p	miR-1249-3p	conserved		16 transcripts	22q13.31	chr22	45200954	45201019	KIAA0930

Intergenic DE microRNAs

DE microRNA	Precursor miRNA	Mature miRNAs	Family	Family Conservation	Clustered miRNAs (10kb from the DE microRNA)	Total no. of predicted targets from Targetscan 7.2	Band	Chromosome	Start	End
hsa-miR-30a-5p	hsa-mir-30a	hsa-miR-30a-5p, hsa-miR-30a-3p	miR-30-5p	broadly conserved	hsa-mir-30d: hsa-mir-30d and hsa-mir-30b	1566 transcripts	6q13	chr6	71403551	71403621
hsa-miR-30d-5p	hsa-mir-30d	hsa-miR-30d-5p, hsa-miR-30d-3p	miR-30-5p	broadly conserved		1566 transcripts	8q24.22	chr8	134804876	134804945
hsa-miR-222-3p	hsa-mir-222	hsa-miR-222-5p, hsa-miR-222-3p	miR-221-3p/222-3p	broadly conserved	hsa-mir-222 and hsa-mir-221	496 transcripts	Xp11.3	chrX	45747015	45747124
hsa-miR-92a-3p	hsa-mir-92a-1	hsa-miR-92a-1-5p, hsa-miR-92a-3p	miR-25-3p/32-5p/92-3p/363-3p/367-3p	broadly conserved	hsa-mir-17, hsa-mir-18a, hsa-mir-19a, hsa-mir-20a, hsa-mir-19b-1, hsa-mir-92a-1	1037 transcripts	13q31.3	chr13	91351314	91351391
hsa-miR-92a-3p	hsa-mir-92a-2	hsa-miR-92a-3p, hsa-miR-92a-2-5p	miR-25-3p/32-5p/92-3p/363-3p/367-3p	broadly conserved	hsa-mir-106a, hsa-mir-18b, hsa-mir-20b, hsa-mir-19b-2, hsa-mir-92a-2, hsa-mir-363	1037 transcripts	Xq26.2	chrX	134169538	134169612

Table S2. Genomic location of DE microRNA transcript from HGNC (Yates et al., 2017) and miRIAD database (Hinske et al., 2014), conservation status, microRNA family information and number of predicted target genes of DE microRNAs from TargetScan Release 7.2 (Agarwal et al., 2015) and microRNA cluster information from miRBase (Kozomara & Griffiths-Jones, 2014) are given.

DE microRNA	Tissue expression data and functions from literature
miR-222	hsa-miR-222-3p is located on Xp11.3 close to Xp11.4, a locus for an X-linked disorder characterised by deafness (Berger et al., 1992) and near to Xp11.23, which shows linkage for non-musical creativity (Oikkonen et al., 2016a). miR-222 is expressed in adult human brain (Burmistrova et al., 2007), human and macaque prefrontal cortex and cerebellum (Hu et al., 2011), mouse brain (Babak et al., 2004), mammalian brains, neurons and cranial ganglia of fore and mid brain (Wienholds et al., 2005). mir-221 (member of mir-222 cluster) is also expressed in mammalian (mouse and rat) primary cortical neurons (Kim et al., 2004) and new born mouse cochlea and vestibule (Friedman et al., 2009; Friedman & Avraham, 2009). miR-222-3p shows differential expression patterns between hippocampus, cerebellum, and prefrontal cortex across various stages of human brain development including early and late childhood and adolescence (Ziats & Rennert, 2014). miR-222 is also differentially expressed between the sensory epithelium of cochlea and vestibule with roles in the regulation of cellular fate during development and in the maintenance of their functions and morphology (Elkan-Miller et al., 2011; Rudnicki et al., 2014). Increased levels of miR-222 was observed during oligodendrocyte maturation (Lau et al., 2008). miR-221, member of the same cluster as the miR-222, is enriched in distal axons (Natera-Naranjo et al., 2010) and expressed during hippocampal neuronal development (Spronsen et al., 2013). miR-222 targets the cell cycle inhibitor *CDKN1B* which encodes p27 protein and shows pro-proliferative functions (Sage et al., 2007; Frenquelli et al., 2010), whereas, in the neuroblastoma cells, miR-222 is activated by p53 and inhibit proliferation (Rihani et al., 2015); hence shows context-dependent functions and represents a switch hub for apoptosis, survival and proliferation; similar to its transcriptional regulator, p53 protein (Rihani et al., 2015). miR-222 is involved in the sensory perception of sound (Zhang et al., 2013). miR-222 is up-regulated in schizophrenia compared to healthy subjects (Perkins et al., 2007) with possible roles in MAPK pathway. In Huntington's disease, a hereditary neurodegenerative disease, miR-222 is found down-regulated in the Brodmann's area 4 (Packer et al., 2008), frontal cortex and striatum (Martí et al., 2010). In AD, miR-222 is found down-regulated in the brain and blood whereas p27 was up-regulated (Wang et al., 2015). miR-221, which belongs to the same cluster as miR-222, is found to be analogous to miR-125a-5p whose increased expression in human brain endothelial cells enhanced the blood brain barrier and helped to form an extensive, continuous junctional complex (Reijerkerk et al., 2013). This is of significance in inflammatory diseases like multiple sclerosis where reduced levels of miR-125a-5p contributed to pro-inflammatory TNFα mediated intracellular cell adhesion molecule (ICAM) and monocyte migration across the blood brain barrier. According to SHIELD database (Shen et al., 2015), miR-222 is expressed in adult cochlear inner and outer hair cells and takes part in cellular response to amino acid stimulus and Wnt receptor signaling pathway. miR-222 was also found down-regulated when exposed to various types of environmental stress inducers including thermal stress (Wilmink et al., 2010) and cigarette smoke (Izzotti et al., 2009).
miR-30d	Located on 8q24.22, near to 8q24 which has been implicated in absolute pitch (Theusch, Basu & Gitschier, 2009), auditory neuropathy (Rance, 2005) and Alzheimer's disease (AD) (Sillén et al., 2011). 8q24 is also the locus for *AGO2* and *MYC*, upstream regulators of the candidate genes in music-performance (Kanduri et al., 2015a), *ARC*, a top candidate for musical ability (Oikkonen et al., 2016b) and *FAM49B*, one of the up-regulated genes from the *high COMB* group (Kanduri et al., 2015b). In music genetic studies, a duplication of the 8q24 region has been noted in individuals with low combined musical score (low *COMB*) (Ukkola-Vuoti et al., 2013). miR-30d is expressed in adult human brain (Burmistrova et al., 2007), prefrontal cortex and cerebellum of adult macaque, chimpanzee and human (Hu et al., 2011), mouse brain (Babak et al., 2004), rat cortical neurons (Kim et al., 2004) and during neuronal development in the hippocampal neurons (Spronsen et al., 2013). miR-30d is expressed in adult cochlear inner and outer hair cells and is involved in cellular response to amino acid stimulus (Shen et al., 2015) and sensory perception of sound (Zhang et al., 2013). miR-30d-5p is amongst the top 40 most expressed microRNA out of the 455 identified in mouse cochlear or vestibular sensory epithelium (Rudnicki et al., 2014) and is also found differentially expressed between hippocampus and cerebellum in adolescent human brain (Ziats & Rennert, 2014). All other members of miR-30 family are also expressed in the cochlear sensory epithelium with significant down-regulation of miR-30d in the cochlea after a day of acoustic over-stimulation or noise exposure and associated sensory cell degeneration (Patel et al., 2013). miR-30a-5p, a cluster member of up-regulated miR-30d is expressed in new borne mouse vestibular sensory epithelium (Friedman et al., 2009; Friedman & Avraham, 2009). miR-30d is increased during oligodendrocyte maturation (Lau et al., 2008) possibly suggestive of its role. miR-30 family, including miR-30d, target *RASA2*, which represses ERk activation in the mouse embryonic stages thereby regulating the ERK signalling pathway that has important functions in cellular homeostasis, cell identity, proliferation and plasticity (Pernaute et al., 2011). hsa-miR-30d-5p is down-regulated in dicer deficient human temporal lobe epileptic hippocampus which might be one of the contributing factors to hippocampal sclerosis (McKiernan et al., 2012). A possible functional role for miR-30 in the visual system is supported by its high expression in Zebra fish lens (Xu, 2009) and in dicer deficient mouse photoreceptor rod (Sundermeier et al., 2014) where it might support rod survival in retinal degeneration. In AD, miR-30d is up-regulated in the CSF (Cogswell et al., 2008) and miR-30e-5p (mir-30 family) is up-regulated in hippocampus (Satoh, 2010). Whereas, miR-30e and miR-30b is found down-regulated in the prefrontal cortex of schizophrenia patients (Perkins et al., 2007).
miR-3909	miR-3909 is located on 22q12.3, whose deletions (22q12.3-q13.1) are associated with neurocristopathy, and sensorineural hearing loss (Jelena et al., 2014). Its preceding region, 2q11.1-.21 is the best linked region for musical aptitude (Oikkonen et al., 2015) and 22q11 is a locus for neuropsychiatric diseases including schizophrenia (Squarcione et al., 2013).
miR-92a	miR-92a-3p is transcribed from 2 locations, Xq26.2 and 13q31. Microdeletion of 13q31.3-q32.1 region has been linked to problems associated with intelligence (Valdes-Miranda et al., 2014). 13q31 is also a locus susceptible for epilepsy (Tauer et al., 2005; EPICURE Consortium et al., 2012), dysplastic ear and psychomotor delays (Grigori et al., 2011). 13q31.3 has been identified as a risk locus for multiple sclerosis (Comabella et al., 2008), a disease of the nervous system characterised by loss of myelin. Expressed in the cochlear and vestibular sensory epithelia (cochlea and vestibule) (Weston et al., 2006; Rudnicki et al., 2014) and in human and mouse brains with roles in neuron differentiation (Sempere et al., 2004). Mammalian miR-92 shows wide expression in most parts of the developing and adult central nervous system with higher intensity in the developing vestibulocochlear ganglion, a region associated with sound perception balance perception from the inner ear signals (Deo et al., 2006). Also expressed in the prefrontal cortex and cerebellum of adult macaque, chimpanzee and human (Hu et al., 2011). Co-expressed along with other cluster members from mir-17 cluster (mir-17, mir-18a, mir-19a, mir-20a, mir-19b-1, hsa-mir-92a) in the developing brain (Miska et al., 2004). miR-92 was profoundly decreased in the brain tissues after traumatic brain injury where a concurrent increase in the β-APP (β-amyloid precursor) and IL-1β are noted (Pinchi et al., 2018).
miR-30a	Expressed in the cochlear and vestibular sensory epithelia (cochlea and vestibule) (Weston et al., 2006; Rudnicki et al., 2014), embryonic and adult cortex, striatum and hippocampus (Landgraf et al., 2007) and developing mammalian brain (Lagos-Quintana et al., 2002) including that of humans with roles in neuronal differentiation (Sempere et al., 2004). miR-30a found to be under the regulation of Dicer and was up-regulated during myelination in the CNS and the PNS (Bremer et al., 2010). The database SHIELD also shows miR-30a expression in adult cochlear inner and outer hair cells (Shen et al., 2015). Also expressed in the prefrontal cortex and cerebellum of adult macaque, chimpanzee and human (Hu et al., 2011). hsa-miR-30a-5p is located on 6q13 in the 6q13-q16.1 region, one of the loci for otosclerosis (OMIM:611572) and sensorineural hearing loss (Thys et al., 2007). It is also located adjacent to *PHIP* (location: 6q14.1), one of the top 40 genes linked to musical abilities from the convergent genomic analysis of musical traits (Oikkonen et al., 2016b). Deletion of 6q13–6q14.1 region has been associated with delays in psychomotor activities and language development (Lespinasse et al., 2009; Mascheretti et al., 2017; Parmeggiani et al., 2017).

Table S3. Candidate microRNAs associated with music-performance of professional musicians, their expression patterns and relevant functional information.

**Comparison**	Target genes of DE microRNAs that show overlap with the compared studies
Target genes of down-regulated microRNAs and singing stimulated genes from songbirds (Wada et al., 2006; Hilliard et al., 2012; Whitney et al., 2014)	*ACTR8, BCL2L13, CEBPD, DOK6, EPDR1, FAF2, FZD7, GGA1, H2AFY2, HMG20B, HNRNPK, LRRC10B, MAFK, MAMLD1, MAPRE2, MLEC, MYO1F, NAB2, OTUD7A, PDCL, PRKRIP1, PTCH1, RAB11FIP4, RAB35, RANBP1, RASD1, SERPINF1, SLC25A25, SLC4A2, SLC6A6, SLMO1, SNPH, SPOPL, SRRM3, STAU2, SYTL4, TGFBRAP1, TMEM104, TRIB2, TRIOBP, TXNRD3, UBE2QL1, WDR26, WDR33, ZFAND6*
Target genes of up-regulated microRNAs and singing inhibited genes from songbirds (Whitney et al., 2014)	*ACACA, AIG1, AJAP1, ANKRD17, ARID4A, ARID4B, ARL5B, ARPP19, ATL2, ATRNL1, ATXN1, B3GALT2, BRMS1L, BTF3L4, CADM2, CAMK2D, CDC42, CNOT1, CRY1, CSMD1, CTCF, CTDSPL2, DBT, DCTN6, DDX3X, DGKI, DIRAS2, DNAJB11, DNAJC21, DUSP6, DYNC1I1, EBAG9, EDEM3, ELK4, ELMO1, EPC1, EXOC6, FAM13C, FAM181B, FARP1, FARSA, FGF14, FKBP1A, FOS, FUBP1, GABRA5, GK, GLB1L2, GRAMD1B, GRM7, HECA, HERC2, HNRNPA3, HNRNPH3, HSPA5, ING5, JHDM1D, JMY, KCTD8, KIAA2026, LRRC10B, MAN1B1, MAPK6, MATR3, MOCS2, MRPL50, MTF1, MYO10, NAA25, NAP1L1, NETO1, NEUROD6, NLGN1, NR2F2, NUS1, PCDH10, PDCD10, PDIA3, PDP2, PLK2, POLR1D, PPP2R2A, R3HDM1, RBM5, RIMS1, RPP30, RWDD1, SAMD12, SCN2A, SERTAD4, SLC15A2, SMC6, SRPR, STXBP5, TAF1, TAGLN3, TBL1XR1, TIMP3, TNRC6B, TOP1, TRIOBP, TULP4, UBXN4, URM1, WDR70, YTHDC1, ZNRF1*

Table S4. Comparative analyses of the target genes of DE microRNAs with singing regulated genes in songbirds.

References

Agarwal V., Bell GW., Nam J-W., Bartel DP. 2015. Predicting effective microRNA target sites in mammalian mRNAs. *Elife* 4. DOI: 10.7554/eLife.05005.

Babak T., Zhang W., Morris Q., Blencowe BJ., Hughes TR. 2004. Probing microRNAs with microarrays: Tissue specificity and functional inference. *RNA* 10:1813–1819. DOI: 10.1261/rna.7119904.

Berger W., Meindl A., Pol TJ van de., Cremers FP., Ropers HH., Döerner C., Monaco A., Bergen AA., Lebo R., Warburg M. 1992. Isolation of a candidate gene for Norrie disease by positional cloning. *Nature Genetics* 1:199–203. DOI: 10.1038/ng0692-199.

Bremer J., O'Connor T., Tiberi C., Rehrauer H., Weis J., Aguzzi A. 2010. Ablation of Dicer from murine Schwann cells increases their proliferation while blocking myelination. *PloS One* 5:e12450. DOI: 10.1371/journal.pone.0012450.

Burmistrova OA., Goltsov AY., Abramova LI., Kaleda VG., Orlova VA., Rogaev EI. 2007. MicroRNA in schizophrenia: Genetic and expression analysis of miR-130b (22q11). *Biochemistry. Biokhimiia* 72:578–582.

Cogswell JP., Ward J., Taylor IA., Waters M., Shi Y., Cannon B., Kelnar K., Kemppainen J., Brown D., Chen C., Prinjha RK., Richardson JC., Saunders AM., Roses AD., Richards CA. 2008. Identification of miRNA changes in Alzheimer's disease brain and CSF yields putative biomarkers and insights into disease pathways. *Journal of Alzheimer's disease: JAD* 14:27–41.

Comabella M., Craig DW., Camiña-Tato M., Morcillo C., Lopez C., Navarro A., Rio J., BiomarkerMS Study Group., Montalban X., Martin R. 2008. Identification of a novel risk locus for multiple sclerosis at 13q31.3 by a pooled genome-wide scan of 500,000 single nucleotide polymorphisms. *PloS One* 3:e3490. DOI: 10.1371/journal.pone.0003490.

Deo M., Yu J-Y., Chung K-H., Tippens M., Turner DL. 2006. Detection of mammalian microRNA expression by in situ hybridization with RNA oligonucleotides. *Developmental Dynamics: An Official Publication of the American Association of Anatomists* 235:2538–2548. DOI: 10.1002/dvdy.20847.

Elkan-Miller T., Ulitsky I., Hertzano R., Rudnicki A., Dror AA., Lenz DR., Elkon R., Irmler M., Beckers J., Shamir R., Avraham KB. 2011. Integration of transcriptomics, proteomics, and microRNA analyses reveals novel microRNA regulation of targets in the mammalian inner ear. *PLoS ONE* 6:e18195. DOI: 10.1371/journal.pone.0018195.

EPICURE Consortium., Leu C., Kovel CGF de., Zara F., Striano P., Pezzella M., Robbiano A., Bianchi A., Bisulli F., Coppola A., Giallonardo AT., Beccaria F., Trenité DK-N., Lindhout D., Gaus V., Schmitz B., Janz D., Weber YG., Becker F., Lerche H., Kleefuss-Lie AA., Hallman K., Kunz WS., Elger CE., Muhle H., Stephani U., Møller RS., Hjalgrim H., Mullen S., Scheffer IE., Berkovic SF., Everett KV., Gardiner MR., Marini C., Guerrini R., Lehesjoki A-E., Siren A.,

Nabbout R., Baulac S., Leguern E., Serratosa JM., Rosenow F., Feucht M., Unterberger I., Covanis A., Suls A., Weckhuysen S., Kaneva R., Caglayan H., Turkdogan D., Baykan B., Bebek N., Ozbek U., Hempelmann A., Schulz H., Rüschendorf F., Trucks H., Nürnberg P., Avanzini G., Koeleman BPC., Sander T. 2012. Genome-wide linkage meta-analysis identifies susceptibility loci at 2q34 and 13q31.3 for genetic generalized epilepsies. *Epilepsia* 53:308–318. DOI: 10.1111/j.1528-1167.2011.03379.x.

Frenquelli M., Muzio M., Scielzo C., Fazi C., Scarfò L., Rossi C., Ferrari G., Ghia P., Caligaris-Cappio F. 2010. MicroRNA and proliferation control in chronic lymphocytic leukemia: Functional relationship between miR-221/222 cluster and p27. *Blood* 115:3949–3959. DOI: 10.1182/blood-2009-11-254656.

Friedman LM., Avraham KB. 2009. MicroRNAs and epigenetic regulation in the mammalian inner ear: Implications for deafness. *Mammalian Genome: Official Journal of the International Mammalian Genome Society* 20:581–603. DOI: 10.1007/s00335-009-9230-5.

Friedman LM., Dror AA., Mor E., Tenne T., Toren G., Satoh T., Biesemeier DJ., Shomron N., Fekete DM., Hornstein E., Avraham KB. 2009. MicroRNAs are essential for development and function of inner ear hair cells in vertebrates. *Proceedings of the National Academy of Sciences of the United States of America* 106:7915–7920. DOI: 10.1073/pnas.0812446106.

Grigori P., Panayiotou E., Sismani C., Koumbaris G., Ioannides M., Costalos C., Kosmaidou-Aravidou Z., Kousoulidou L., Patsalis PC. 2011. 21 Mb deletion in chromosome band 13q22.2q32.1 associated with mild/moderate psychomotor retardation, growth hormone insufficiency, short neck, micrognathia, hypotonia, dysplastic ears and other dysmorphic features. *European Journal of Medical Genetics* 54:365–368. DOI: 10.1016/j.ejmg.2011.02.006.

Hilliard AT., Miller JE., Fraley ER., Horvath S., White SA. 2012. Molecular microcircuitry underlies functional specification in a basal ganglia circuit dedicated to vocal learning. *Neuron* 73:537–552. DOI: 10.1016/j.neuron.2012.01.005.

Hinske LC., França GS., Torres HAM., Ohara DT., Lopes-Ramos CM., Heyn J., Reis LFL., Ohno-Machado L., Kreth S., Galante PAF. 2014. miRIAD-integrating microRNA inter- and intragenic data. *Database (Oxford)* 2014. DOI: 10.1093/database/bau099.

Hu HY., Guo S., Xi J., Yan Z., Fu N., Zhang X., Menzel C., Liang H., Yang H., Zhao M., Zeng R., Chen W., Pääbo S., Khaitovich P. 2011. MicroRNA expression and regulation in human, chimpanzee, and macaque brains. *PLoS genetics* 7:e1002327. DOI: 10.1371/journal.pgen.1002327.

Izzotti A., Calin GA., Arrigo P., Steele VE., Croce CM., De Flora S. 2009. Downregulation of microRNA expression in the lungs of rats exposed to cigarette smoke. *FASEB journal: official publication of the Federation of American Societies for Experimental Biology* 23:806–812. DOI: 10.1096/fj.08-121384.

Jelena B., Christina L., Eric V., Fabiola Q-R. 2014. Phenotypic variability in Waardenburg syndrome resulting from a 22q12.3-q13.1 microdeletion involving SOX10. *American Journal of Medical Genetics. Part A* 164A:1512–1519. DOI: 10.1002/ajmg.a.36446.

Kanduri C., Kuusi T., Ahvenainen M., Philips AK., Lähdesmäki H., Järvelä I. 2015a. The effect of music performance on the transcriptome of professional musicians. *Scientific Reports* 5:9506. DOI: 10.1038/srep09506.

Kanduri C., Raijas P., Ahvenainen M., Philips AK., Ukkola-Vuoti L., Lähdesmäki H., Järvelä I. 2015b. The effect of listening to music on human transcriptome. *PeerJ* 3:e830. DOI: 10.7717/peerj.830.

Kim J., Krichevsky A., Grad Y., Hayes GD., Kosik KS., Church GM., Ruvkun G. 2004. Identification of many microRNAs that copurify with polyribosomes in mammalian neurons. *Proceedings of the National Academy of Sciences of the United States of America* 101:360–365. DOI: 10.1073/pnas.2333854100.

Kozomara A., Griffiths-Jones S. 2014. miRBase: Annotating high confidence microRNAs using deep sequencing data. *Nucleic Acids Research* 42:D68–73. DOI: 10.1093/nar/gkt1181.

Lagos-Quintana M., Rauhut R., Yalcin A., Meyer J., Lendeckel W., Tuschl T. 2002. Identification of tissue-specific microRNAs from mouse. *Current biology: CB* 12:735–739.

Landgraf P., Rusu M., Sheridan R., Sewer A., Iovino N., Aravin A., Pfeffer S., Rice A., Kamphorst AO., Landthaler M., Lin C., Socci ND., Hermida L., Fulci V., Chiaretti S., Foà R., Schliwka J., Fuchs U., Novosel A., Müller R-U., Schermer B., Bissels U., Inman J., Phan Q., Chien M., Weir DB., Choksi R., De Vita G., Frezzetti D., Trompeter H-I., Hornung V., Teng G., Hartmann G., Palkovits M., Di Lauro R., Wernet P., Macino G., Rogler CE., Nagle JW., Ju J., Papavasiliou FN., Benzing T., Lichter P., Tam W., Brownstein MJ., Bosio A., Borkhardt A., Russo JJ., Sander C., Zavolan M., Tuschl T. 2007. A mammalian microRNA expression atlas based on small RNA library sequencing. *Cell* 129:1401–1414. DOI: 10.1016/j.cell.2007.04.040.

Lau P., Verrier JD., Nielsen JA., Johnson KR., Notterpek L., Hudson LD. 2008. Identification of dynamically regulated microRNA and mRNA networks in developing oligodendrocytes. *The Journal of Neuroscience: The Official Journal of the Society for Neuroscience* 28:11720–11730. DOI: 10.1523/JNEUROSCI.1932-08.2008.

Lespinasse J., Gimelli S., Béna F., Antonarakis SE., Ansermet F., Paoloni-Giacobino A. 2009. Characterization of an interstitial deletion 6q13-q14.1 in a female with mild mental retardation, language delay and minor dysmorphisms. *European Journal of Medical Genetics* 52:49–52. DOI: 10.1016/j.ejmg.2008.10.001.

Love MI., Huber W., Anders S. 2014. Moderated estimation of fold change and dispersion for RNA-seq data with DESeq2. *Genome Biology* 15:550. DOI: 10.1186/s13059-014-0550-8.

Martí E., Pantano L., Bañez-Coronel M., Llorens F., Miñones-Moyano E., Porta S., Sumoy L., Ferrer I., Estivill X. 2010. A myriad of miRNA variants in control and Huntington's disease brain

regions detected by massively parallel sequencing. *Nucleic Acids Research* 38:7219–7235. DOI: 10.1093/nar/gkq575.

Mascheretti S., De Luca A., Trezzi V., Peruzzo D., Nordio A., Marino C., Arrigoni F. 2017. Neurogenetics of developmental dyslexia: From genes to behavior through brain neuroimaging and cognitive and sensorial mechanisms. *Translational Psychiatry* 7:e987. DOI: 10.1038/tp.2016.240.

McKiernan RC., Jimenez-Mateos EM., Bray I., Engel T., Brennan GP., Sano T., Michalak Z., Moran C., Delanty N., Farrell M., O'Brien D., Meller R., Simon RP., Stallings RL., Henshall DC. 2012. Reduced mature microRNA levels in association with dicer loss in human temporal lobe epilepsy with hippocampal sclerosis. *PLoS ONE* 7:e35921. DOI: 10.1371/journal.pone.0035921.

Miska EA., Alvarez-Saavedra E., Townsend M., Yoshii A., Sestan N., Rakic P., Constantine-Paton M., Horvitz HR. 2004. Microarray analysis of microRNA expression in the developing mammalian brain. *Genome Biology* 5:R68. DOI: 10.1186/gb-2004-5-9-r68.

Natera-Naranjo O., Aschrafi A., Gioio AE., Kaplan BB. 2010. Identification and quantitative analyses of microRNAs located in the distal axons of sympathetic neurons. *RNA* 16:1516–1529. DOI: 10.1261/rna.1833310.

Oikkonen J., Huang Y., Onkamo P., Ukkola-Vuoti L., Raijas P., Karma K., Vieland VJ., Järvelä I. 2015. A genome-wide linkage and association study of musical aptitude identifies loci containing genes related to inner ear development and neurocognitive functions. *Molecular Psychiatry* 20:275–282. DOI: 10.1038/mp.2014.8.

Oikkonen J., Kuusi T., Peltonen P., Raijas P., Ukkola-Vuoti L., Karma K., Onkamo P., Järvelä I. 2016a. Creative Activities in Music - A Genome-Wide Linkage Analysis. *PLoS ONE* 11:e0148679. DOI: 10.1371/journal.pone.0148679.

Oikkonen J., Onkamo P., Järvelä I., Kanduri C. 2016b. Convergent evidence for the molecular basis of musical traits. *Scientific Reports* 6:39707. DOI: 10.1038/srep39707.

Packer AN., Xing Y., Harper SQ., Jones L., Davidson BL. 2008. The bifunctional microRNA miR-9/miR-9* regulates REST and CoREST and is downregulated in Huntington's disease. *The Journal of Neuroscience: The Official Journal of the Society for Neuroscience* 28:14341–14346. DOI: 10.1523/JNEUROSCI.2390-08.2008.

Parmeggiani G., Bigoni S., Buldrini B., Garani G., Clauser L., Galiè M., Ferlini A., Fini S. 2017. Double Interstitial Deletion of the Long Arm of Chromosome 6 in a Patient with Pierre Robin Sequence, Dysmorphisms, and Severe Developmental Delay. *Molecular Syndromology* 9:30–37. DOI: 10.1159/000480159.

Patel M., Cai Q., Ding D., Salvi R., Hu Z., Hu BH. 2013. The miR-183/Taok1 target pair is implicated in cochlear responses to acoustic trauma. *PLoS ONE* 8:e58471. DOI: 10.1371/journal.pone.0058471.

Perkins DO., Jeffries CD., Jarskog LF., Thomson JM., Woods K., Newman MA., Parker JS., Jin J., Hammond SM. 2007. microRNA expression in the prefrontal cortex of individuals with schizophrenia and schizoaffective disorder. *Genome Biology* 8:R27. DOI: 10.1186/gb-2007-8-2-r27.

Pernaute B., Spruce T., Rodriguez TA., Manzanares M. 2011. MiRNA-mediated regulation of cell signaling and homeostasis in the early mouse embryo. *Cell Cycle (Georgetown, Tex.)* 10:584–591. DOI: 10.4161/cc.10.4.14728.

Pinchi E., Frati A., Cipolloni L., Aromatario M., Gatto V., La Russa R., Pesce A., Santurro A., Fraschetti F., Frati P., Fineschi V. 2018. Clinical-pathological study on β-APP, IL-1β, GFAP, NFL, Spectrin II, 8OHdG, TUNEL, miR-21, miR-16, miR-92 expressions to verify DAI-diagnosis, grade and prognosis. *Scientific Reports* 8:2387. DOI: 10.1038/s41598-018-20699-1.

Rance G. 2005. Auditory neuropathy/dys-synchrony and its perceptual consequences. *Trends in Amplification* 9:1–43.

Reijerkerk A., Lopez-Ramirez MA., Het Hof B van., Drexhage JAR., Kamphuis WW., Kooij G., Vos JB., Pouw Kraan TCTM van der., Zonneveld AJ van., Horrevoets AJ., Prat A., Romero IA., Vries HE de. 2013. MicroRNAs regulate human brain endothelial cell-barrier function in inflammation: Implications for multiple sclerosis. *The Journal of Neuroscience: The Official Journal of the Society for Neuroscience* 33:6857–6863. DOI: 10.1523/JNEUROSCI.3965-12.2013.

Rihani A., Van Goethem A., Ongenaert M., De Brouwer S., Volders P-J., Agarwal S., De Preter K., Mestdagh P., Shohet J., Speleman F., Vandesompele J., Van Maerken T. 2015. Genome wide expression profiling of p53 regulated miRNAs in neuroblastoma. *Scientific Reports* 5:9027. DOI: 10.1038/srep09027.

Rudnicki A., Isakov O., Ushakov K., Shivatzki S., Weiss I., Friedman LM., Shomron N., Avraham KB. 2014. Next-generation sequencing of small RNAs from inner ear sensory epithelium identifies microRNAs and defines regulatory pathways. *BMC Genomics* 15:484. DOI: 10.1186/1471-2164-15-484.

Sage C le., Nagel R., Egan DA., Schrier M., Mesman E., Mangiola A., Anile C., Maira G., Mercatelli N., Ciafrè SA., Farace MG., Agami R. 2007. Regulation of the p27(Kip1) tumor suppressor by miR-221 and miR-222 promotes cancer cell proliferation. *The EMBO journal* 26:3699–3708. DOI: 10.1038/sj.emboj.7601790.

Satoh J-i. 2010. MicroRNAs and their therapeutic potential for human diseases: Aberrant microRNA expression in Alzheimer's disease brains. *Journal of Pharmacological Sciences* 114:269–275.

Sempere LF., Freemantle S., Pitha-Rowe I., Moss E., Dmitrovsky E., Ambros V. 2004. Expression profiling of mammalian microRNAs uncovers a subset of brain-expressed microRNAs with possible roles in murine and human neuronal differentiation. *Genome Biology* 5:R13. DOI: 10.1186/gb-2004-5-3-r13.

Shen J., Scheffer DI., Kwan KY., Corey DP. 2015. SHIELD: An integrative gene expression database for inner ear research. *Database: The Journal of Biological Databases and Curation* 2015:bav071. DOI: 10.1093/database/bav071.

Sillén A., Brohede J., Forsell C., Lilius L., Andrade J., Odeberg J., Kimura T., Winblad B., Graff C. 2011. Linkage analysis of autopsy-confirmed familial Alzheimer disease supports an Alzheimer disease locus in 8q24. *Dementia and Geriatric Cognitive Disorders* 31:109–118. DOI: 10.1159/000323808.

Spronsen M van., Battum EY van., Kuijpers M., Vangoor VR., Rietman ML., Pothof J., Gumy LF., Ijcken WFJ van., Akhmanova A., Pasterkamp RJ., Hoogenraad CC. 2013. Developmental and activity-dependent miRNA expression profiling in primary hippocampal neuron cultures. *PLoS ONE* 8:e74907. DOI: 10.1371/journal.pone.0074907.

Squarcione C., Torti MC., Di Fabio F., Biondi M. 2013. 22q11 deletion syndrome: A review of the neuropsychiatric features and their neurobiological basis. *Neuropsychiatric Disease and Treatment* 9:1873–1884. DOI: 10.2147/NDT.S52188.

Sundermeier TR., Zhang N., Vinberg F., Mustafi D., Kohno H., Golczak M., Bai X., Maeda A., Kefalov VJ., Palczewski K. 2014. DICER1 is essential for survival of postmitotic rod photoreceptor cells in mice. *FASEB journal: official publication of the Federation of American Societies for Experimental Biology* 28:3780–3791. DOI: 10.1096/fj.14-254292.

Tauer U., Lorenz S., Lenzen KP., Heils A., Muhle H., Gresch M., Neubauer BA., Waltz S., Rudolf G., Mattheisen M., Strauch K., Nürnberg P., Schmitz B., Stephani U., Sander T. 2005. Genetic dissection of photosensitivity and its relation to idiopathic generalized epilepsy. *Annals of Neurology* 57:866–873. DOI: 10.1002/ana.20500.

Theusch E., Basu A., Gitschier J. 2009. Genome-wide study of families with absolute pitch reveals linkage to 8q24.21 and locus heterogeneity. *American Journal of Human Genetics* 85:112–119. DOI: 10.1016/j.ajhg.2009.06.010.

Thys M., Van Den Bogaert K., Iliadou V., Vanderstraeten K., Dieltjens N., Schrauwen I., Chen W., Eleftheriades N., Grigoriadou M., Pauw RJ., Cremers CRWJ., Smith RJH., Petersen MB., Van Camp G. 2007. A seventh locus for otosclerosis, OTSC7, maps to chromosome 6q13-16.1. *European journal of human genetics: EJHG* 15:362–368. DOI: 10.1038/sj.ejhg.5201761.

Ukkola-Vuoti L., Kanduri C., Oikkonen J., Buck G., Blancher C., Raijas P., Karma K., Lähdesmäki H., Järvelä I. 2013. Genome-Wide Copy Number Variation Analysis in Extended Families and Unrelated Individuals Characterized for Musical Aptitude and Creativity in Music. *PLoS ONE* 8:e56356. DOI: 10.1371/journal.pone.0056356.

Valdes-Miranda JM., Soto-Alvarez JR., Toral-Lopez J., González-Huerta L., Perez-Cabrera A., Gonzalez-Monfil G., Messina-Bass O., Cuevas-Covarrubias S. 2014. A novel microdeletion involving the 13q31.3-q32.1 region in a patient with normal intelligence. *European Journal of Medical Genetics* 57:60–64. DOI: 10.1016/j.ejmg.2014.01.006.

Wada K., Howard JT., McConnell P., Whitney O., Lints T., Rivas MV., Horita H., Patterson MA., White SA., Scharff C., Haesler S., Zhao S., Sakaguchi H., Hagiwara M., Shiraki T., Hirozane-Kishikawa T., Skene P., Hayashizaki Y., Carninci P., Jarvis ED. 2006. A molecular neuroethological approach for identifying and characterizing a cascade of behaviorally regulated genes. *Proceedings of the National Academy of Sciences of the United States of America* 103:15212–15217. DOI: 10.1073/pnas.0607098103.

Wang X., Xu Y., Zhu H., Ma C., Dai X., Qin C. 2015. Downregulated microRNA-222 is correlated with increased p27Kip[1] expression in a double transgenic mouse model of Alzheimer's disease. *Molecular Medicine Reports* 12:7687–7692. DOI: 10.3892/mmr.2015.4339.

Weston MD., Pierce ML., Rocha-Sanchez S., Beisel KW., Soukup GA. 2006. MicroRNA gene expression in the mouse inner ear. *Brain Research* 1111:95–104. DOI: 10.1016/j.brainres.2006.07.006.

Whitney O., Pfenning AR., Howard JT., Blatti CA., Liu F., Ward JM., Wang R., Audet J-N., Kellis M., Mukherjee S., Sinha S., Hartemink AJ., West AE., Jarvis ED. 2014. Core and region-enriched networks of behaviorally regulated genes and the singing genome. *Science (New York, N.Y.)* 346:1256780. DOI: 10.1126/science.1256780.

Wienholds E., Kloosterman WP., Miska E., Alvarez-Saavedra E., Berezikov E., Bruijn E de., Horvitz HR., Kauppinen S., Plasterk RHA. 2005. MicroRNA expression in zebrafish embryonic development. *Science* 309:310–311. DOI: 10.1126/science.1114519.

Wilmink GJ., Roth CL., Ibey BL., Ketchum N., Bernhard J., Cerna CZ., Roach WP. 2010. Identification of microRNAs associated with hyperthermia-induced cellular stress response. *Cell Stress & Chaperones* 15:1027–1038. DOI: 10.1007/s12192-010-0189-7.

Xu S. 2009. microRNA expression in the eyes and their significance in relation to functions. *Progress in Retinal and Eye Research* 28:87–116. DOI: 10.1016/j.preteyeres.2008.11.003.

Yates B., Braschi B., Gray KA., Seal RL., Tweedie S., Bruford EA. 2017. Genenames.org: The HGNC and VGNC resources in 2017. *Nucleic Acids Research* 45:D619–D625. DOI: 10.1093/nar/gkw1033.

Zhang Q., Liu H., McGee J., Walsh EJ., Soukup GA., He DZZ. 2013. Identifying microRNAs involved in degeneration of the organ of corti during age-related hearing loss. *PLoS ONE* 8:e62786. DOI: 10.1371/journal.pone.0062786.

Ziats MN., Rennert OM. 2014. Identification of Differentially Expressed MicroRNAs Across the Developing Human Brain. *Molecular psychiatry* 19:848–852. DOI: 10.1038/mp.2013.93.
