# Supplementary material for: Music-performance regulates microRNAs in professional musicians
Source: PeerJ. 2019 Mar 29;7:e6660. doi: 10.7717/peerj.6660 (PMC6442922; doi:10.7717/peerj.6660)
Supplement: Supplemental Information 5 [file peerj-07-6660-s005.pdf]

Music-performance and Control study

The venue of the music-performance under study and the principal concert venue of *Tapiola Sinfonietta* is Tapiola Hall, which has a capacity of approximately 800 seats with high-quality equipments and professional support staff. *Tapiola Sinfonietta* is the official orchestra of the Espoo city and is housed in Espoo Cultural Centre, Finland where other cultural activities are regularly organised. All the musicians who took part in the music-performance have played together for many years at the same venue (Tapiola Hall) and have also performed together during their regular tours for concert performances at international venues. The classical symphony concert we studied was performed after rehearsals under the guidance of a conductor, who used physical gestures during the concert-performance towards interpretation of the music pieces for fine-tuned performance. Whereas, the control study was organised at the Sibelius Academy, University of Arts, Helsinki in a music-free environment (without playing or listening to music) during which some professional musicians chose to listen to a lecture and some went for a gentle walk outside for 2 hours, approximately the same duration as the music-performance.

Sample collection, RNA extraction and sequencing

Peripheral whole blood samples (2.5 ml) were collected from the musicians immediately before and immediately after the music-performance and control study into PAXgene Blood RNA tubes (PreAnalytiX GmbH, Hombrechtikon, Switzerland) and stored at -20°C until processing. RNA was isolated with the PAXgene Blood microRNA Kit (PreAnalytiX GmbH, Hombrechtikon, Switzerland), concentrated, mixed, incubated at -80°C for 30 minutes and then centrifuged at >12,000 x g for 30 minutes at 4°C. Next, the supernatant was removed; samples were washed and incubated for 10 minutes on ice, then RNA pellet was re-suspended in an appropriate amount of RNase-free water (Applied Biosystems, USA). Samples were then measured for concentration and purity on the NanoDrop 1000 v.3.7 (Thermo Fisher Scientific, USA) and for integrity on the 2100 Bioanalyzer (Agilent Technologies, Germany). Small RNA libraries were prepared at the high throughput genomics department of The Wellcome Trust Centre for Human Genetics at the Oxford University using the NEBNext® Small RNA Library Prep Set for Illumina (Multiplex Compatible) and sequenced with Illumina (Illumina Inc., USA) HiSeq.
